# The US‐Based DIAMOND Lewy Toolkit: Overview and management of motor symptoms

**DOI:** 10.1002/alz.090803

**Published:** 2025-01-09

**Authors:** James B Leverenz, John T O'Brien, John‐Paul Taylor

**Affiliations:** ^1^ Cleveland Clinic, Cleveland, OH USA; ^2^ Department of Psychiatry, University of Cambridge, Cambridge United Kingdom; ^3^ Newcastle University, Newcastle upon Tyne United Kingdom

## Abstract

Lewy body dementia (LBD) clinically is characerized by a complex myriad of cognitive, behavioral, motor, sleep and autonomic symptoms that can be challenging to manage, particularly in the context of the fluctuations that are so characteristic for these patients. The DIAMOND Lewy Toolkits offer a resource to support clinicians in recognizing and managing this range of symptoms observed in LBD. This session will provide a guided introduction to the DIAMOND Lewy Toolkits, offering a framework to leverage the toolkits in support of patient centered care. In addition to an overview of the DIAMOND Lewy program (Dr. Leverenz), he will focus on motor symptom management. Subsequent speakers will address neuropsychiatric and sleep symptoms (Dr. Chiadi), cognition and autonomic dysfunction (Dr. Scharre) and the adaptation of the DIAMOND Lewy Tool kits in the U.S. (Dr. Amodeo).

